# Impact of COVID-19 on out-of-hospital cardiac arrest care processes

**DOI:** 10.12968/jpar.2023.15.2.74

**Published:** 2023-02-08

**Authors:** Betty Pennington, Steve Bell, Adam Wright, James E Hill

**Affiliations:** 1 Research Paramedic; North West Ambulance Service NHS Trust; 2 Consultant Paramedic; North West Ambulance Service NHS Trust; 3 Senior Research Fellow, University of Central Lancashire, Colne, Lancashire; UK

**Keywords:** Out-of-hospital cardiac arrest, COVID-19, Systematic review, Automated external defibrillators, Resuscitation

## Abstract

Early bystander cardiopulmonary resuscitation, use of defibrillators (including automated external defibrillators) and timely treatment by emergency medical services are known to increase the chances of survival for a patient experiencing an out-of-hospital cardiac arrest (OHCA); however, the impact of the COVID-19 pandemic on this is unclear from examining previous literature. This commentary critically appraises a recent systematic review and meta-analysis which assesses the effect of the COVID-19 pandemic on pre-hospital care for OHCA.

## Introduction

A variety of factors influence the clinical outcome of an out-of-hospital cardiac arrest (OHCA), including the initiation of bystander cardiopulmonary resuscitation (BCPR), the accessibility and time to defibrillation, including the use of automated external defibrillators (AEDs) and treatment by pre-hospital medical services (Thannhauser et al, 2022). The immediacy of such interventions is paramount to improve the chances of survival. OHCA patients who receive BCPR are twice as likely to survive to discharge, whilst early defibrillation by public AEDs is associated with a 68% increase in survival to discharge rates (CARES, 2018). Previous studies have examined the impact of the coronavirus disease 2019 (COVID-19) pandemic on the components of the “chain of survival”. Whilst the hesitancy of bystanders to perform CPR during the pandemic has been documented, conflicting results have been found in rates of BCPR in OHCA occurring in the pandemic (Grunau et al, 2020, Fazel et al, 2022). It also remains unclear how the strain of the pandemic on emergency medical services (EMS) influenced pre-hospital care processes, including resuscitation practices and the impact of adhering to Personal Protective Equipment (PPE) requirements (Lim et al, 2020). A recent systematic review by Masuda et al (2022) aimed to provide clarity on the effect of the COVID-19 pandemic on the “chain of survival” and determine to what extent OHCA care processes were disrupted.

### Aim of commentary

This commentary aims to critically appraise the methods used within the review by Masuda et al (2022) and expand upon the findings in the context of clinical practice.

## Methods

The systematic review and meta-analysis were reported in accordance to the Preferred Reporting Items for Systematic Reviews and Meta-Analyses (PRISMA) guidance. A comprehensive literature search was undertaken with a restricted date range from the first reported COVID-19 case (31^st^ December 2019) to 3^rd^ May 2021 on five bibliographic databases (PubMed, EMBASE, Web of Science, Scopus and The Cochrane Central Register for Controlled Trials). Articles were included that discussed OHCA during the pandemic and compared outcomes during and before the pandemic. Articles lacking a historical control and those discussing fewer than five patients were excluded. Screening, data extraction and methodological quality of included studies (Newcastle-Ottawa Scale [NOS]) was carried out independently by three authors. Meta-analyses were conducted for both the community and EMS processes. A random-effects model was utilised to estimate the effects of COVID-19 due to significant between-study heterogeneity.

## Results

The search strategy identified 966 records. After full-screening, 20 observational, cross-sectional studies, that compared the COVID-19 pandemic period with historical control data, were included. The studies were from 10 different countries across Asia, Australia, Europe and the United States of America. The risk of bias was deemed to be low and the quality high for all included papers, based on assessment using the Newcastle-Ottawa Scale. The key findings from the review were that the odds of a patient having an OHCA at home were significantly higher during the pandemic than before the pandemic (OR 1.38, 95% CI 1.11-1.71), whilst the odds of EMS resuscitation being attempted (OR 0.84, 95% CI 0.73-0.97) or an AED being applied to a patient experiencing OHCA (OR 0.65, 95% CI 0.48-0.88) were both significantly lower during the pandemic than before it. Whilst endotracheal intubation significantly decreased (OR 0.48, 95% CI 0.27-0.85), the odds of a supraglottic airway being used conversely significantly increased (OR 2.04, 95% CI 1.09-3.82) during the pandemic period. Furthermore, it took significantly longer from time of EMS call to EMS crews reaching patients in OHCA during the pandemic than pre-pandemic (SMD 0.27, 95% CI 0.13-0.40).

There was no evidence that the witness rates of OHCA, BCPR rates, resuscitation duration, administration of amiodarone and epinephrine rates and use of mechanical CPR were different before and during the pandemic. Considerable heterogeneity was found in all the above results, with the exception of EMS resuscitation attempted, which had substantial heterogeneity.

Additional leave-one out analyses, and meta-regression and sub-group analyses for BCPR all revealed no statistically significant changes to the findings. BCPR was also used to determine the absence of publication bias by examining both a funnel plot, which revealed no asymmetry, and through Egger’s regression test, which was non-significant.

### Commentary

This systematic review and meta-analysis aimed to investigate and synthesise the effect of the COVID-19 pandemic on OHCA care and to articulate the impact of the pandemic on the processes of pre-hospital care delivery associated with the chain of survival through consideration of the current evidence base. Critical appraisal of the methods used to undertake the review using the Joanna Briggs Institute (JBI) Critical Appraisal Tool for Systematic Reviews (JBI, 2020) reveals a high methodological standard with all criteria achieved demonstrating a robust process. The completeness and high-quality approach to the methodology instils confidence that this review provides a comprehensive summary, and contextualisation of the published evidence on the topic. In investigating the impact of the COVID-19 pandemic on the pre-hospital chain of survival, this review is distinguished from previous reviews undertaken in providing a clear focus upon the factors deemed to have direct, demonstrable impact upon survival from OHCA; in this context the review question is clearly defined with the inclusion and exclusion criteria appropriately set.

Whilst the findings from this review are contextualised by being specific to occurrences during the midst of the COVID-19 pandemic, the results and subsequent impacts upon considerations for system-level care delivery are of interest to those involved in pre-hospital resuscitation for OHCA. Brady et al (2022) describe a ‘burden’ placed upon pre-hospital providers delivery of cardiac arrest care owing to the impacts of the COVID-19 pandemic and describe that, despite multiple efforts, there was adverse impact upon the chain of survival. This systematic review provides high quality evidence of those impacts which may be of use to inform future pandemic protocols whether COVID related or otherwise. Such considerations may include protocols associated with pre-arrival care, the time impacts on the delivery of critical interventions associated with increased demand and considerations such as PPE donning and the availability of medical devices and procedures viewed in the context of the prevalent disease (Ball et al, 2020).

Given the nature of social restrictions in place during the pandemic, including ‘stay at home’ orders, travel restrictions and lockdowns (Girum et al, 2021), the finding that there was a significant increase in OHCA occurring at home is perhaps not surprising. It is reassuring to observe that there was no difference in BCPR rates during the pandemic as compared to before. However, the finding that there was a significant decrease in AED use during the COVID-19 pandemic is alarming given the empirically proven benefit of defibrillation as a vital link in the chain of survival which directly links to positive outcomes (Brooks et al, 2022). Again, this is not necessarily surprising given the findings around the location of OHCA and an increase in private rather than public locations where AEDs tend to be situated. This finding, together with those attributed to EMS processes which included a significant decrease in attempted EMS resuscitation and a significant increase in EMS call to arrival times, demonstrates the value of this review in informing the future design and delivery of pre-hospital OHCA care in both pandemic and non-pandemic times. The findings emphasise the need to target the accessibility and availability of EMS services and AEDs to incidents of OHCA, including the need to maximize access to AEDs from individual homes rather than a focus upon their accessibility within public spaces. This is equally applicable in both pandemic and non-pandemic times with this review highlighting potential gaps in service provision within EMS and within the community. In addressing the findings of this review there is the opportunity to improve the pre-hospital chain of survival and ultimately the outcomes from OHCA.

Future research in this area should explore if the impacts of the pandemic demonstrated in this review are long lasting. If so, further development of specific interventions targeting these particular effects are required. As there was a wide range of strategies used across the world during the pandemic, future research should explore which restrictions/changing services are important mediating factors on the delivery of OHCA care, with the aim to reflect upon these findings and make recommendations for future pandemic protocol development.

### CPD reflective questions

- Do you know where the nearest public access AED is to your home address and if not, how might you find this out? Is this information that you could share with patients/members of the public?
- Is there anything you could safely do to reduce the amount of time taken to reach a patient experiencing an OHCA, particularly during a pandemic when there may be a requirement to put on additional personal protective equipment before approaching the patient?
- Did your airway management change for patients in cardiac arrest during the COVID-19 pandemic? If so, why? And was this appropriate for your patient, and for yourself?

## Key Points

- This high-quality systematic review and meta-analysis, looking at community and ambulance care processes for out-of-hospital cardiac arrest, found several significant differences during the COVID-19 pandemic, compared to before the pandemic
- During the pandemic there were longer times for emergency medical services to reach patients in out-of-hospital cardiac arrest, and lower odds of both emergency medical services staff attempting resuscitation and of an automated external defibrillator being used
- Improvement in public access to automated external defibrillators from residential settings may be beneficial to improve out-of-hospital cardiac arrest outcomes
